# *c-MYC* Copy-Number Gain Is an Independent Prognostic Factor in Patients with Colorectal Cancer

**DOI:** 10.1371/journal.pone.0139727

**Published:** 2015-10-01

**Authors:** Kyu Sang Lee, Yoonjin Kwak, Kyung Han Nam, Duck-Woo Kim, Sung-Bum Kang, Gheeyoung Choe, Woo Ho Kim, Hye Seung Lee

**Affiliations:** 1 Department of Pathology, Seoul National University Bundang Hospital, Seongnam-si, Republic of Korea; 2 Department of Pathology, Seoul National University College of Medicine, Seoul, Republic of Korea; 3 Department of Pathology, Haeundae Paik Hospital, Inje University College of Medicine, Busan, Republic of Korea; 4 Department of Surgery, Seoul National University Bundang Hospital, Seongnam-si, Republic of Korea; Heinrich-Heine-University and University Hospital Duesseldorf, GERMANY

## Abstract

**Background:**

The aim of this study was to determine the incidence and clinicopathological significance of *c-MYC* gene copy-number (GCN) gain in patients with primary colorectal cancer (CRC).

**Methods:**

The *c-MYC* GCN was investigated in 367 consecutive CRC patients (cohort 1) by using dual-color silver *in situ* hybridization. Additionally, to evaluate regional heterogeneity, we examined CRC tissue from 3 sites including the primary cancer, distant metastasis, and lymph-node metastasis in 152 advanced CRC patients (cohort 2). *KRAS* exons 2 and 3 were investigated for mutations.

**Results:**

In cohort 1, *c-MYC* gene amplification, defined by a *c-MYC*:centromere of chromosome 8 ratio ≥ 2.0, was detected in 31 (8.4%) of 367 patients. A *c-MYC* GCN gain, defined by ≥ 4.0 *c-MYC* copies/nucleus, was found in 63 (17.2%) patients and was associated with poor prognosis (*P* = 0.015). Multivariate Cox regression analysis showed that the hazard ratio for *c-MYC* GCN gain was 2.35 (95% confidence interval, 1.453–3.802; *P* < 0.001). In a subgroup of stage II-III CRC patients, *c-MYC* GCN gain was significantly associated with poor prognosis by univariate (*P* = 0.034) and multivariate (*P* = 0.040) analyses. *c-MYC* protein overexpression was observed in 201 (54.8%) out of 367 patients and weakly correlated with *c-MYC* GCN gain (ρ, 0.211). In cohort 2, the *c-MYC* genetic status was heterogenous in advanced CRC patients. Discordance between GCN gain in the primary tumor and either distant or lymph-node metastasis was 25.7% and 30.4%, respectively. A similar frequency for *c-MYC* GCN gain and amplification was observed in CRC patients with both wild-type and mutated *KRAS*.

**Conclusions:**

*c-MYC* GCN gain was an independent factor for poor prognosis in consecutive CRC patients and in the stage II-III subgroup. Our findings indicate that the status of *c-MYC* may be helpful in predicting the patients’ outcome and for managing CRC patients.

## Introduction

The *c-MYC* proto-oncogene encodes a transcription factor that plays a central role in cell proliferation, differentiation, apoptosis, metabolism, and survival [1, 2]. It can promote tumorigenesis in a variety of human malignancies [3, 4]. *c-MYC* alteration occurs through various mechanisms, including chromosomal translocation, gene amplification, and perturbation of upstream signaling pathways [5, 6]. Gene copy-number (GCN) gain or amplification is the most common *c-MYC* alteration in solid tumors [7].

Nevertheless, few studies have examined the clinicopathological implications of *c-MYC* status in colorectal cancer (CRC). Previous reports have shown that *c-MYC* GCN gain in CRC is found in approximately 10% of patients [8]. A recent study reported that several significant amplifications were focused on chromosome 8, including the 8q24 region which contains *c-MYC*, and suggested that *c-MYC* was a new marker for aggressive disease in CRC [9]. However, more recently, Christopher *et al*. reported data obtained by immunohistochemistry (IHC), indicating that c-MYC protein overexpression was significantly associated with improved prognosis in CRC patients [10]. Consequently, the prognostic value of *c-MYC* alterations in CRC is controversial.

Recently, the range of options for systemic chemotherapy has expanded and targeted therapy has been used in advanced CRC patients, increasing patient survival [11]. However, some CRC patients respond poorly to targeted therapy despite showing positive results in targeted therapy-specific mutation studies [12]. Tumor heterogeneity is a potential cause for failure of targeted therapy and several studies have reported that CRC possess a heterogenic genotype including *KRAS*, *p53*, and *BRAF* [13–15]. Therefore, genetic variation between the primary tumor and corresponding metastatic sites needs to be clarified to improve the management of CRC patients with metastatic disease.

The heterogeneity of *c-MYC* and its prognostic implications have not been systematically studied in primary CRC patients. The aim of this study was to evaluate *c-MYC* gene status and its clinical significance in CRC. We also analyzed the heterogeneity of *c-MYC* in the primary tumor and distant metastasis.

## Materials and Methods

### Patients and samples

A total of 519 CRC patients treated with radical surgery at Seoul National University Bundang Hospital were enrolled in this retrospective study. First, to evaluate the clinicopathologic significance of *c-MYC* gene status, 367 consecutive CRC patients treated between January 2005 and December 2006 were enrolled (cohort 1). Second, to investigate the discordance between the primary and metastatic tumors, 152 advanced CRC patients with synchronous or metachronous metastasis who had undergone surgical resection for primary CRC between May 2003 and December 2009, were enrolled (cohort 2). All the cases were reviewed by two pathologists (K. S. L. and H. S. L.). The clinicopathological characteristics were obtained from the patients’ medical records and pathology reports. Follow-up information including patient outcome and the interval between the date of surgical resection and death was collected. Data from patients lost to follow-up or those who had died from causes other than CRC were censored.

### Ethical statement

All samples were obtained from surgically resected tumors examined pathologically at the Department of Pathology, Seoul National University Bundang Hospital. All samples and medical record data were anonymized before use in this study and the participants did not provide written informed consent. The use of medical record data and tissue samples for this study was approved by the Institutional Review Board of Seoul National University Bundang Hospital (reference: B-1210/174-301).

### Tissue array method

Surgically resected primary CRC specimens were formalin-fixed and paraffin-embedded (FFPE). For each case, representative areas of the donor blocks were obtained and rearranged into new recipient blocks (Superbiochips Laboratories, Seoul, South Korea) [16]. A single core was 2 mm in diameter and those containing > 20% tumor cells were considered valid cores.

### Dual-color silver *in situ* hybridization

**Fig 1:**
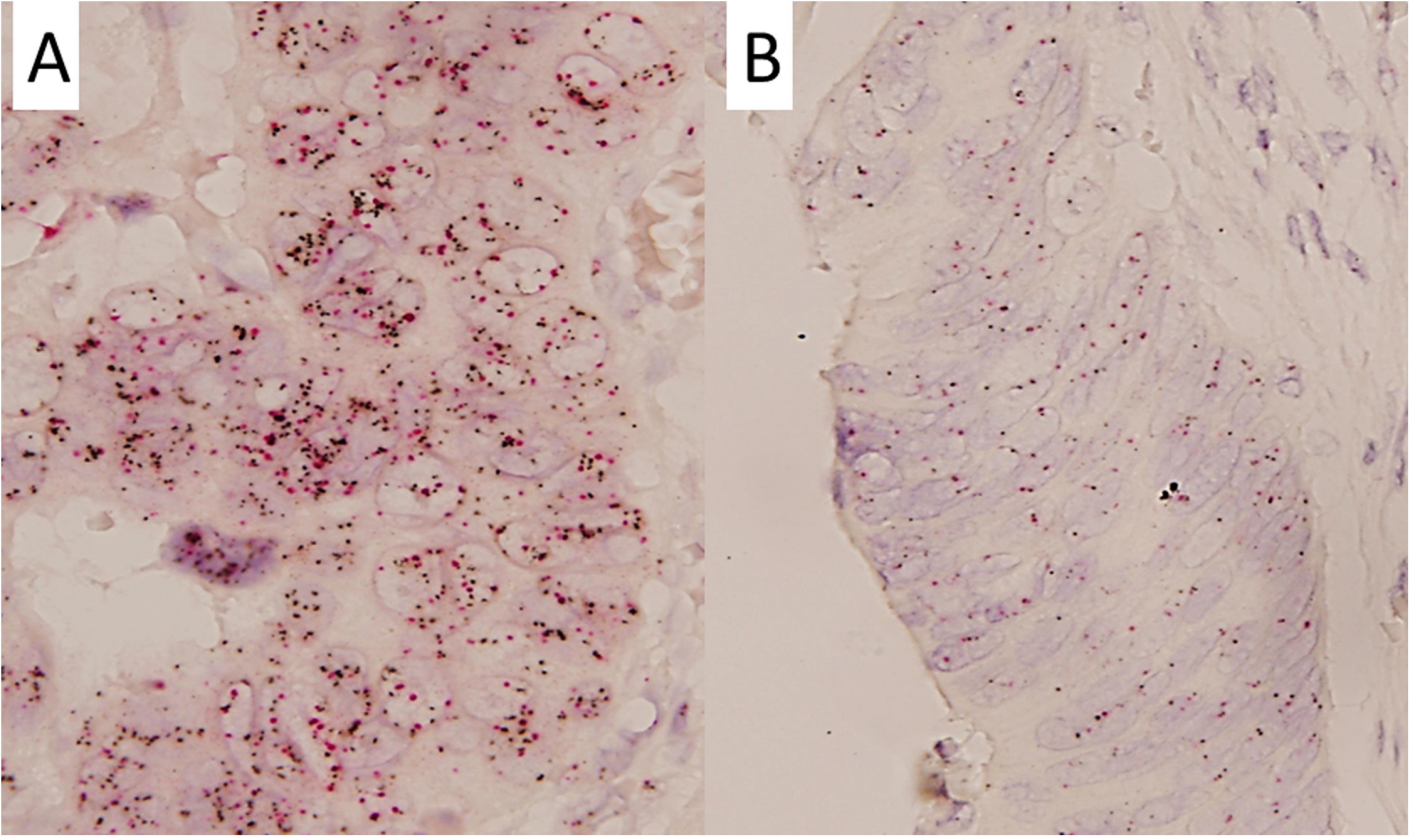
Representative figures of *c-MYC* status detected by dual-color silver *in situ* hybridization (A and B) in colorectal cancer patients. (A) *c-MYC* gene copy number gain (60 × magnification); (B) *c-MYC* gene disomy (60 × magnification).

The *c-MYC* gene was visualized by using a blue-staining system (ultraView silver *in situ* hybridization [SISH] dinitrophenol [DNP] detection kit and c-MYC DNP probe, Ventana Medical Systems, Tucson, AZ, USA). The centromere of chromosome 8 (CEP8) was visualized by using a red-staining system (ultraView red ISH digoxigenin [DIG] detection kit and chromosome 8 DIG probe, Ventana Medical Systems). Positive signals were visualized at 60 × magnification and counted in 50 non-overlapping tumor cell nuclei for each case (Fig 1) [17]. Small and large clusters were scored as 6 and 12 signals, respectively.

### Immunohistochemistry

IHC analysis of c-MYC was carried out using a commercially available rabbit anti-c-MYC antibody (clone Y69, catalog ab32072, Abcam, Burlingame, CA, USA). The staining procedures were carried out using the ultraView Universal DAB kit (Ventana Medical Systems) and an automated stainer (BenchMark®XT, Ventana Medical Systems), according to the manufacturer’s instructions. Nuclear immunostaining of c-MYC was negative in normal mucosa. For statistical analysis, c-MYC nuclear staining of any intensity in greater than 10% of neoplastic cells was scored as positive (S2 Fig) [10].

### Microsatellite instability

Microsatellite instability (MSI) was assessed in CRC cases with available tissue. MSI results were generated by comparing the allelic profiles of 5 microsatellite markers (BAT-26, BAT-25, D5S346, D17S250, and S2S123) in the tumor and corresponding normal samples. Polymerase chain reaction (PCR) products from the FFPE tissues were analyzed using an automated DNA sequencer (ABI 3731 Genetic Analyser, Applied Bio systems, Foster City, CA, USA) according to the protocol described previously [18].

### *KRAS* mutation analysis

*KRAS* mutation detection was achieved by melting curve analysis using the cobas 4800 System (Roche, Branchburg, NJ, USA) with automated result interpretation software. This is a TaqMelt-based real-time PCR assay designed to detect the presence of 21 *KRAS* mutations in codons 12, 13, and 61. The workflow and testing process have been described previously [19].

### Statistical analyses

The association between the clinicopathological features and *c-MYC* status was analyzed using the chi-square or Fisher’s exact test, as appropriate. The correlation between the detection methods was examined using the Pearson correlation coefficient. The patients’ survival was analyzed by using the Kaplan-Meier method and the log-rank test was used to determine if there were any significant differences between the survival curves. Univariate and multivariate regression analysis were performed by using Cox’s proportional hazards model to determine the hazard ratio and 95% confidence intervals for each factor. A *P-*value < 0.05 was accepted as statistically significant. All statistical analyses were performed using the SPSS statistics 21 software (IBM, Armonk, NY, USA).

## Results

### *c-MYC* gene status and clinical implications for consecutive primary CRC patients

**Fig 2:**
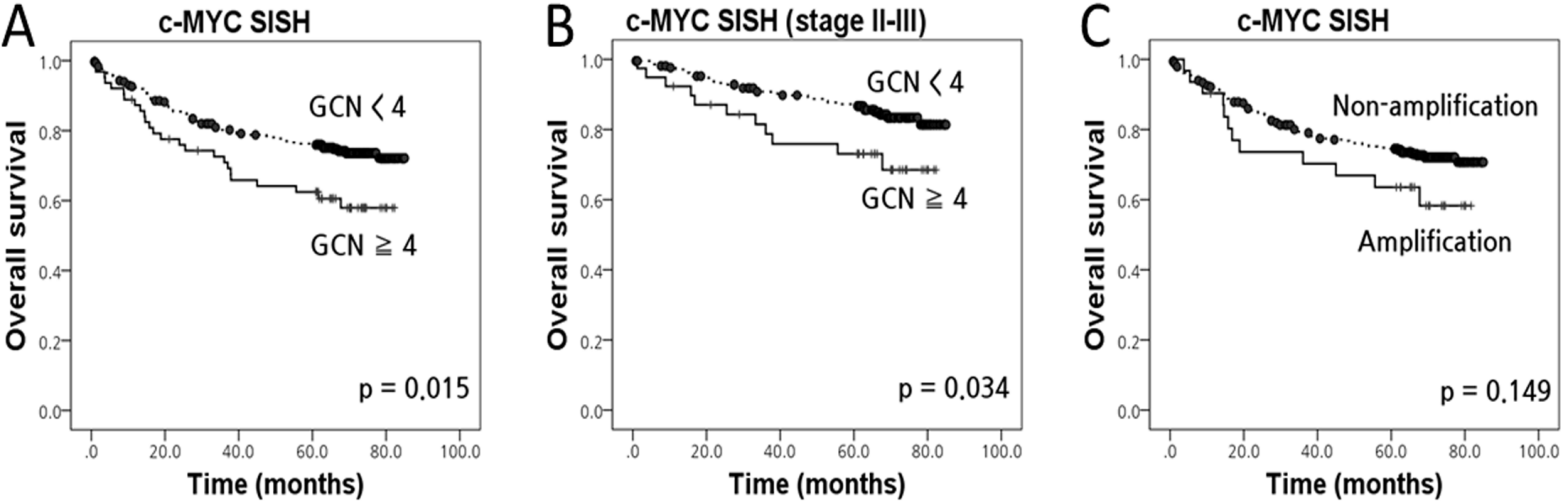
Kaplan-Meier survival curves illustrating the prognostic effect of *c-MYC* status in colorectal cancer (cohort 1). (A) *c-MYC* gene copy number (GCN) gain; (B) *c-MYC* GCN gain in the stage II-III subgroup; (C) *c-MYC* amplification.

In consecutive primary CRC cases (cohort 1), the median *c-MYC*:CEP8 ratio was 1.29 (range, 0.58–5.17). *c-MYC* gene amplification, defined by a *c-MYC*:CEP8 ratio ≥ 2.0 and similar to that established for *HER2* [20], was detected in 31 (8.4%) of 367 patients. The mean *c-MYC* GCN was 2.88 (range, 1.22–13.12). In the present study, we defined the GCN gain as ≥ 4.0 *c-MYC* copies/nucleus [21], and this was detected in 63 (17.2%) of 367 CRC patients. All *c-MYC* amplification was included in *c-MYC* GCN gain. A *c-MYC* GCN gain ≥ 4 had the lowest *P*-value (*P* = 0.015) and thus, was observed to be the most predictive cut-off point for patient prognosis (Fig 2); ≥ 5.0 *c-MYC* copies/nucleus also influenced patient prognosis (*P* = 0.026). There was no significant association between patient prognosis and either *c-MYC* amplification (*P* = 0.149) or > 2, ≥ 3, and ≥ 6 *c-MYC* copies/nucleus (*P* = 0.752, *P* = 0.175, and *P* = 0.122, respectively).

Table 1 shows the relationships between *c-MYC* status and the clinicopathological parameters in consecutive primary CRCs (cohort 1). Amplification of *c-MYC* correlated with early-stage disease (*P* = 0.039). *c-MYC* GCN gain was frequently observed in sigmoid colon and rectum tumors (*P* = 0.034), small tumors (*P* = 0.041), and those classified as microsatellite stable or MSI-low (*P* = 0.029).

**Table 1:** The association between clinicopathological parameters and *c-MYC* status in 367 CRC patients (cohort1).

	Total	c-Myc		*P*-Value	c-Myc		*P*-Value	c-Myc IHC		*P*-Value
		4 > GCN	4 ≦ GCN		Non-amplification	Amplification		Negative	Positive	
Age				0.983			0.383			0.537
mean	64.2	64.2	64.2		64.1	66.0		64.6	63.9	
Sex				0.740			0.619			0.431
male	205	171 (83.4%)	34 (16.6%)		189 (92.2%)	16 (7.8%)		89 (43.4%)	116 (56.6%)	
female	162	133 (82.1%)	29 (17.9%)		147 (90.7%)	15 (9.3%)		77 (47.5%)	85 (52.5%)	
Location				**0.034**			0.437			**< 0.001**
Rectum/sigmoid	237	189 (79.7%)	48 (20.3%)		121 (93.1%)	9 (6.9%)		90 (38.0%)	147 (62.0%)	
others	130	115 (88.5%)	15 (11.5%)		215 (90.7%)	22 (9.3%)		76 (58.5%)	54 (41.5%)	
pT stage				0.692			0.571			**< 0.001**
0–2	58	47 (81.0%)	11 (19.0%)		52 (89.7%)	6 (10.3%)		14 (24.1%)	44 (75.9%)	
3–4	309	257 (83.2%)	52 (16.8%)		284 (91.9%)	25 (8.1%)		152 (49.2%)	157 (50.8%)	
Differentiation				0.139			0.055			**0.007**
LG	331	271 (81.9%)	60 (18.1%)		300 (90.6%)	31 (9.4%)		142 (42.9%)	189 (57.1%)	
HG	36	33 (91.7%)	3 (8.3%)		36 (100.0%)	0 (0.0%)		24 (66.7%)	12 (33.3%)	
LN metastasis				0.609			0.070			0.058
absent	168	141 (83.9%)	27 (16.1%)		149 (88.7%)	19 (11.3%)		67 (39.9%)	101 (60.1%)	
present	199	163 (81.9%)	36 (18.1%)		187 (94.0%)	12 (6.0%)		99 (49.7%)	100 (50.3%)	
Lymphatic invasion				0.152			0.896			0.073
absent	158	136 (86.1%)	22 (13.9%)		145 (91.8%)	13 (8.2%)		63 (39.9%)	95 (60.1%)	
present	209	168 (80.4%)	41 (19.6%)		191 (91.4%)	18 (8.6%)		103 (49.3%)	106 (50.7%)	
Perineural invasion				0.631			0.530			**0.025**
absent	154	212 (83.5%)	42 (16.5%)		231 (90.9%)	23 (9.1%)		49 (58.7%)	105 (41.3%)	
present	113	92 (81.4%)	21 (18.6%)		105 (92.9%)	8 (7.1%)		61 (54.0%)	52 (46.0%)	
Venous invasion				0.776			0.999			0.814
absent	296	246 (83.1%)	50 (16.9%)		271 (91.6%)	25 (8.4%)		133 (44.9%)	163 (55.1%)	
present	71	58 (81.7%)	13 (18.3%)		65 (91.5%)	6 (8.5%)		33 (46.5%)	38 (53.5%)	
Tumor border				0.524			0.327			0.544
expanding	60	48 (80.0%)	12 (20.0%)		53 (88.3%)	7 (11.7%)		25 (41.7%)	35 (58.3%)	
infiltrative	307	256 (83.4%)	51 (16.6%)		283 (92.2%)	24 (7.8%)		141 (45.9)	166 (54.1%)	
Size (cm)				**0.041**			0.061			**< 0.001**
mean	5.3	5.4	4.7		5.3	4.5		5.8	4.8	
Distant metastasis				0.123			0.544			0.252
absent	299	252 (84.3%)	47 (15.7%)		275 (92.0%)	24 (8.0%)		131 (43.8%)	168 (56.2%)	
present	68	52 (76.5%)	16 (23.5%)		61 (89.7%)	7 (10.3%)		35 (51.5%)	33 (48.5%)	
pTNM stage				0.822			**0.039**			0.050
I, II	162	135 (83.0%)	27 (17.0%)		140 (88.1%)	19 (11.9%)		64 (39.5%)	98 (60.5%)	
III, IV	205	169 (82.4%)	36 (17.6%)		193 (94.1%)	12 (5.9%)		102 (49.8%)	103 (50.2%)	
MSI status				**0.029**			0.256			0.490
MSS/MSI-L	323	264 (81.7%)	59 (18.3%)		294 (91.0%)	29 (9.0%)		141 (38.4%)	182 (49.6%)	
MSI-H	32	31 (96.9%)	1 (3.1%)		31 (96.9%)	1 (3.1%)		16 (1.6%)	16 (4.4%)	

Abbreviations: CRC, colorectal cancer; T, tumor; LG, low grade; HG, high grade; LN, lymph node; MSS, microsatellite stable; MSI-L, microsatellite instability-low; MSI-H, microsatellite instability-high; GCN, gene copy number; IHC, immunohistochemistry

*P*-values are calculated by using χ^2^-test or Fisher’s exact test

### Prognostic significance of *c-MYC* gene status in CRC patients

All CRC patients were successfully followed up for inclusion in the survival analysis (Fig 2). In cohort 1, the mean follow-up period was 55 months (range, 1–85 months) and 101 (27.5%) patients died during the follow-up period. Kaplan-Meier analysis showed that *c-MYC* GCN gain was significantly associated with poor survival in CRC patients (*P* = 0.015), but *c-MYC* amplification was not (*P* = 0.149). In the stage II-III subgroup, *c-MYC*-GCN gain also predicted poor prognosis (*P* = 0.034). Multivariate analysis of *c-MYC* status is summarized in Table 2, and showed that *c-MYC*-GCN gain independently predicted poor prognosis in the consecutive cohort (*P* < 0.001) and in the subgroup of patients with stage II-III CRC (*P* = 0.040).

**Table 2:** Multivariate Cox proportional hazard models for the predictors of overall survival (cohort 1).

	Univariate survival analysis		Multivariate survival analysis	
Factors	HR (95% CI)	*P* value	HR (95% CI)	*P* value
**c-MYC GCN SISH (4≦ vs. 4>)**	**1.756 (1.117–2.763)**	**0.015**	**2.350 (1.453–3.801)**	**<0.001**
Age	1.026 (1.008–1.045)	0.005	1.025 (1.007–1.043)	0.006
Size	1.244 (1.059–1.244)	0.001	1.099 (0.995–1.214)	NS (0.062)
Histologic grade (high vs. low)	3.143 (1.904–5.188)	<0.001	2.844 (1.625–4.977)	<0.001
Stage (3/4 vs. 1/2)	6.151 (3.494–10.829)	<0.001	3.069 (1.603–5.878)	0.001
Lymphatic invasion	3.661 (2.242–5.980)	<0.001	1.251 (0.709–2.205)	NS (0.439)
Perineural invasion	3.942 (2.648–5.870)	<0.001	2.325 (1.487–3.636)	<0.001
Venous invasion	3.985 (2.671–5.946)	<0.001	2.304 (1.490–3.676)	<0.001
**c-MYC GCN SISH (4≦ vs. 4>) in a subgroup of stage II/III**	**2.057 (1.039–4.073)**	**0.038**	**2.058 (1.032–4.105)**	**0.040**
Age	1.037 (1.009–1.067)	0.010	1.036 (1.007–1.066)	0.014
Stage (3 vs. 2)	2.955 (1.493–5.850)	0.002	1.705 (0.802–3.623)	NS (0.165)
Lymphatic invasion	2.882 (1.456–5.703)	0.002	1.846 (0.887–3.845)	NS (0.101)
Perineural invasion	3.536 (1.952–6.405)	0.001	2.921 (1.558–5.476)	<0.001

Abbreviations: SISH, silver in-situ hybridization; GCN, gene copy number; HR, hazard ratio

*P*-values are calculated by using χ^2^-test or Fisher’s exact test

### Correlation between the *c-MYC* GCN gain and protein overexpression

Overexpression of c-MYC protein was detected in 201 (54.8%) of 367 CRC patients (cohort 1) and was associated with early pT stage (*P* < 0.001), low grade of histologic differentiation (*P* = 0.007), absence of perineural invasion (*P* = 0.025) and smaller size (*P* < 0.001) (Table 1). Overexpression of c-MYC protein was associated with GCN gain (ρ, 0.211; *P* < 0.001), which was categorized as weakly correlation according to Dancey and Reidy’s categorization (2004) [22]. Furthermore, only 46 (22.9%) of 201 patients with c-MYC overexpression showed a GCN gain.

### *c-MYC* status and heterogeneity according to tumor location in advanced CRC patients

To evaluate the regional heterogeneity of *c-MYC* status, we examined tissue from 3 sites including the primary cancer, distant metastasis, and lymph-node metastasis for each patient with advanced CRC (cohort 2). In the primary tumors of cohort 2, the median *c-MYC*:CEP8 ratio was 1.14 (range, 0.57–2.97). *c-MYC* gene amplification was detected in 8 (5.3%) of 152 patients. The mean *c-MYC* GCN was 2.97 (range, 1.40–9.94). *c-MYC* GCN gain was detected in 48 (31.6%) of 152 CRC patients. In addition, *c-MYC* GCN gain was found in 33 (21.7%) patients with distant metastatic tumors. Lymph-node metastasis was observed in 79 of 152 advanced CRC patients and *c-MYC* GCN gain was observed in 18 (22.8%) of these cases. The heterogeneity of *c-MYC* GCN gain according to tumor location is shown in Table 3. Of 152 cases, discordance between *c-MYC* GCN gain in the primary tumor and distant metastasis was noted in 39 (25.7%) cases. Discordance between *c-MYC* GCN gain in the primary tumor and lymph-node metastasis was detected in 24/79 (30.4%) cases. Thus, regional heterogeneity of *c-MYC* GCN gain was quite common in advanced CRC. *c-MYC* GCN heterogeneity was not correlated with clinicopathological factors and prognosis (*P* > 0.05; data not shown).

**Table 3:** Heterogeneity of *c-MYC* GCN gain with respect to tumor location in advanced CRC (cohort 2).

c-MYC GCN gain (%)		Primary		
		negative	positive	total
Distant metastasis	negative	92 (60.5)	27 (17.8)	152 (100)
	positive	12 (7.9)	21 (13.8)	
LN metastasis	negative	44 (55.7)	17 (21.5)	79 (100)
	positive	7 (8.9)	11 (13.9)	

Abbreviations: GCN, gene copy number; LN: lymph node

*P*-values are calculated by using χ^2^-test or Fisher’s exact test

There was no statistically significant correlation between the clinicopathological factors and *c-MYC* GCN gain in primary, distant metastatic, and lymph-node metastatic tumors from cohort 2 CRC patients (*P* > 0.05; data not shown). The mean follow-up time was 42 months (range, 1–105 months) and 67 patients (44.1%) died from cancer during the follow-up period. Kaplan-Meier analysis showed that patients with *c-MYC* GCN gain in the primary tumor had a poor outcome than those without, but this result was not statistically significant (*P* = 0.499). However, ≥ 3.0 *c-MYC* copies/nucleus in the primary tumor was significantly associated with a poor prognosis (*P* = 0.044; S1 Fig). There was no significant correlation between the patients’ prognosis and *c-MYC* GCN gain in distant or lymph-node metastases (*P* = 0.981 and *P* = 0.417, respectively; data not shown).

### *KRAS* mutations in advanced CRC

The cobas *KRAS* test was performed on 152 primary tumors from advanced CRC cases (cohort 2). *KRAS* gene mutations were observed in 84 (55.3%) cases and were associated with tumors located in the right colon (*P* = 0.019), but were not correlated with other clinicopathological factors (*P* > 0.05; data not shown). Additionally, there was no statistical difference between the survival of CRC patients with mutated or wild-type *KRAS* (*P* = 0.688; data not shown). Of 68 cases with wild-type *KRAS*, *c-MYC* amplification was noted in 4 (5.9%) and a *c-MYC* GCN gain in 28 (41.2%). Of 84 cases with mutated *KRAS*, 4 showed *c-MYC* amplification (4.8%) and 20 (23.8%) revealed a *c-MYC* GCN gain. *c-MYC* GCN alterations occurred in patients with both wild-type and mutated KRAS. Therefore, *c-MYC* GCN alterations and *KRAS* mutations were not mutually exclusive.

## Discussion

Although there have been several reports on *c-MYC* status in human cancers, there are no established criteria for GCN gain. Cancers with a *c-MYC* GCN gain are often associated with a poor prognosis. A previous study of mucinous gastric carcinoma showed that *c-MYC* amplification, defined as a *c-MYC*:CEP8 ratio > 2.0, was strongly correlated with the advanced stages of cancer [23]. Another report found an association between *c-MYC* amplification (> 4 copies/cell in a minimum of 10% of tumor cells) and the advanced stages of ovarian cancer [21]. In a study of prostate cancer, the *c-MYC* GCN gain included the criterion of a *c-MYC/*CEP8 ratio > 1.5, and a poor prognosis was observed for patients in this category [24]. In recent research on adenocarcinoma of the lung, patients with > 2 *c-MYC* copies/nucleus were classified as having an increased *c-MYC* GCN, which was found to be an independent poor prognostic factor [25]. In CRC patients, it was reported that *c-MYC* amplification, defined as a *c-MYC/*CEP8 ratio > 2, was frequently detected by using fluorescent *in situ* hybridization (9.0–14.2%), but was unrelated to clinical outcome and pathological data [26]. Therefore, we have applied diverse criteria to determine *c-MYC* amplification or GCN gain in this study, and have defined the *c-MYC* GCN gain as ≥ 4 copies/nucleus, because it had the lowest *P*-value for disease prognosis (Fig 2). In cohort 1, the large consecutive cohort, CRC patients with a *c-MYC* GCN gain had a poor survival than those without (*P* = 0.015). Furthermore, in multivariate analysis, *c-MYC* GCN gain was a significant CRC prognostic factor, both in the consecutive cohort and for those with stage II-III disease. The predictive value of the *c-MYC* GCN was found to be independent of known prognostic factors. The *c-MYC* GCN gain criteria used in the present study, together with the SISH method, may be useful in assessing CRC patients because it clearly identified patients expected to have poor survival, regardless of the *c-MYC*:CEP8 ratio.

In cohort 2, we showed that there was *c-MYC* GCN regional heterogeneity between the primary site and its related metastases. A *c-MYC* GCN gain (c-MYC GCN ≥ 4.0) in the primary cancer was not significantly associated with poor survival (*P* = 0.499; S1 Fig), which might be because all of cohort 2 consisted of advanced CRC patients with synchronous and metachronous metastasis and cohort 2 was largely comprised of stage IV CRC (98 cases; 64.5%). They received a variety of personalized treatment respectively and these might reflect the statistical insignificance. Interestingly, we applied slightly non-restrictive criteria of GCN gain (*c-MYC* GCN ≥ 3.0) and its prognosis was changed to statistically significant (*P* = 0.044; S1 Fig). In a broad sense, c-*MYC* GCN gain of primary cancer tends to correlated with poor survival in advanced CRC. On the other hand, *c-MYC* status in distant and lymph-node metastatic lesion was not related to patient prognosis although we tried every possible GCN criteria. Even though, *c-MYC* heterogeneity was observed frequently in advanced CRC, a *c-MYC* GCN gain in the primary cancer was often associated with poor survival. Consequently, the *c-MYC* GCN in the non-metastatic lesion should be used when evaluating prognosis.

In a previous study, overexpression of *c-MYC* mRNA in CRC was found to be associated with a better prognosis [27], but this result was contradicted by another study [28]. Christopher *et al*. recently demonstrated that c-MYC overexpression determined by IHC alone, was significantly associated with a better survival in CRC patients when assessed by univariate analysis, but not by multivariate analysis [10]. Interestingly, we found conflicting results in a previous c-MYC overexpression study; presumably, because c-MYC expression is controlled by a complex regulatory pathway involving multiple interactions with other molecules, rather than just simple GCN gain [29]. Furthermore, we found a weak correlation between c-MYC protein overexpression and GCN gain in CRC patients. *c-MYC* GCN gain was not observed in most c-MYC protein overexpression cases. Unlike the *c-MYC* GCN gain, overexpression of c-MYC protein was correlated with less aggressive features (Table 1). These results suggest that *c-MYC* GCN gain is probably only partly responsible for protein overexpression. As overexpression of c-MYC is not the same as a *c-MYC* GCN gain, further research is needed to explain the difference of c-MYC overexpression and GCN gain in CRC tumorigenesis.

Mutations in *KRAS* are evident in 30–40% of colorectal tumors [30–32]. Indeed, previous studies reported that a *KRAS* mutation was associated with resistance to anti-epidermal growth factor receptor (EGFR) monoclonal therapy and a poor survival [33–35]. In our study, *KRAS* mutations were present in 55.3% of advanced CRC patients (cohort 2) and were not associated with prognosis. It may be because we investigated *KRAS* mutation status in advanced CRC patients. Phipps et al. also reported that *KRAS*-mutation status was not associated with poor disease specific survival in cases who presented with distant-stage CRC [33]. *c-MYC* amplification was observed in 5.9% of wild-type *KRAS* and 4.8% of mutated *KRAS* CRCs. A *c-MYC* GCN gain was observed in 41.2% of wild-type *KRAS* and 23.8% of mutated *KRAS* CRCs. It is noteworthy that these 2 genetic events were not mutually exclusive. Further studies are required to investigate the possibility of using *c-MYC* genetic alterations as therapeutic targets in advanced CRC patients with primary and secondary resistance to anti-EGFR therapies.

In summary, we comprehensively analyzed the *c-MYC* gene status of CRC patients by using SISH. *c-MYC* GCN gain and amplification were observed in 17.2% and 8.4% of consecutive CRC patients, respectively. The *c-MYC* GCN gain was an independent poor prognostic factor, both in the consecutive cohort and in the subgroup of patients with stage II-III disease. These findings show that *c-MYC* status can be used to predict the prognosis of CRC patients, and may inform future studies on the pathogenesis and mechanisms involved in the progression of CRC.

## Supporting Information

S1 Fig Kaplan-Meier survival curves illustrating the prognostic effect of *c-MYC* status in primary lesions of colorectal cancer (cohort 2). (A) *c-MYC* gene copy number (GCN) ≥ 3.0; (B) *c-MYC* GCN ≥ 4.0.
(TIF)

S2 Fig Representative figures of c-MYC overexpression by immunohistochemistry (A and B) in colorectal cancer patients. (A) c-MYC overexpression (40 × magnification); (B) No c-MYC expression (40 × magnification);
(TIF)
