# Supplementary figures and images for: c-MYC Copy-Number Gain Is an Independent Prognostic Factor in Patients with Colorectal Cancer
Source: PLoS One. 2015 Oct 1;10(10):e0139727. doi: 10.1371/journal.pone.0139727 (PMC4591346; doi:10.1371/journal.pone.0139727)

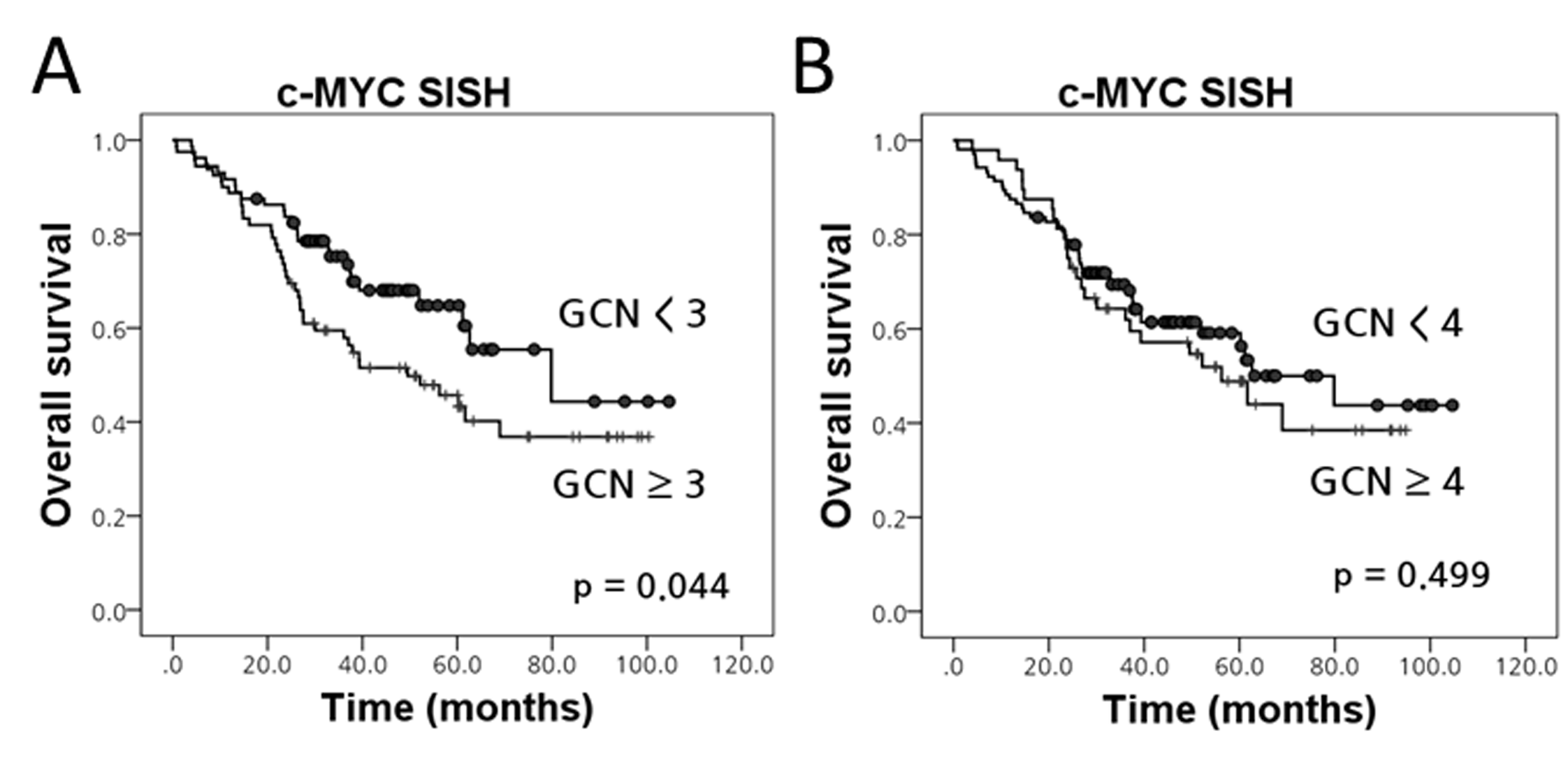

Supplement: S1 Fig — (A) c-MYC gene copy number (GCN) ≥ 3.0; (B) c-MYC GCN ≥ 4.0. (TIF) [file pone.0139727.s001.tif]

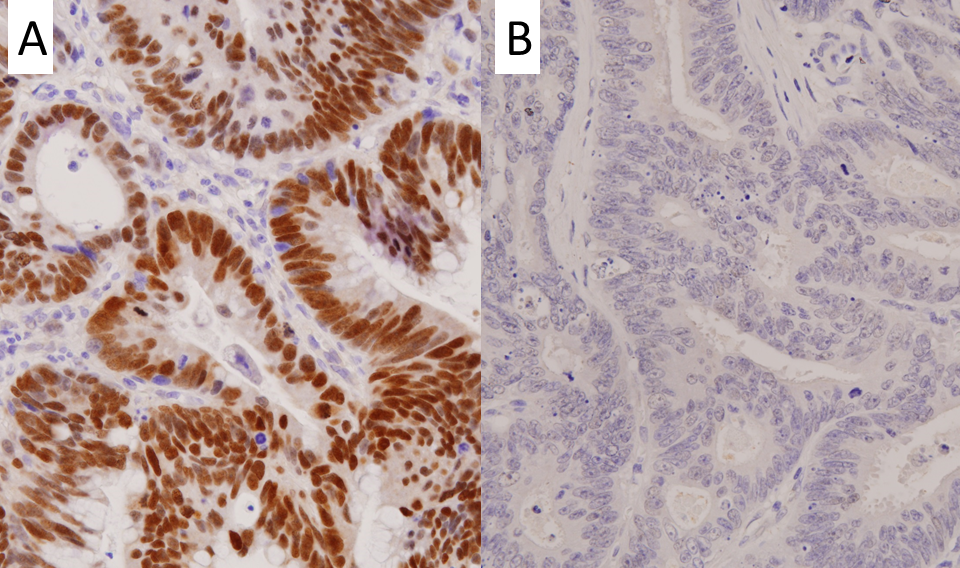

Supplement: S2 Fig — (A) c-MYC overexpression (40 × magnification); (B) No c-MYC expression (40 × magnification); (TIF) [file pone.0139727.s002.tif]
